# Glucose tolerance test with a single abnormal value as a predictor of type 2 diabetes mellitus: a multicenter retrospective study

**DOI:** 10.1038/s41598-024-57535-8

**Published:** 2024-03-21

**Authors:** Seon Ui Lee, Subeen Hong, Sae Kyung Choi, Su Mi Kim, Jae Eun Shin, Ki Cheol Kil, Yeon Hee Kim, Jeong Ha Wie, Yun Sung Jo, Hyun Sun Ko

**Affiliations:** 1 Department of Obstetrics and Gynecology, College of Medicine, Incheon St. Mary’s Hospital, The Catholic University of Korea, Seoul, Korea; 2 Department of Obstetrics and Gynecology, Seoul St. Mary’s Hospital, College of Medicine, The Catholic University of Korea, 222 Banpo-daero, Seocho-Gu, Seoul, 06591 Korea; 3 Department of Obstetrics and Gynecology, Daejeon St. Mary’s Hospital, College of Medicine, The Catholic University of Korea, Seoul, Korea; 4 Department of Obstetrics and Gynecology, Bucheon St. Mary’s Hospital, College of Medicine, The Catholic University of Korea, Seoul, Korea; 5 Department of Obstetrics and Gynecology, Yeouido St. Mary’s Hospital, College of Medicine, The Catholic University of Korea, Seoul, Korea; 6 Department of Obstetrics and Gynecology, Uijeongbu St. Mary’s Hospital, College of Medicine, The Catholic University of Korea, Seoul, Korea; 7 Department of Obstetrics and Gynecology, Eunpyeong St. Mary’s Hospital, College of Medicine, The Catholic University of Korea, Seoul, Korea; 8 Department of Obstetrics and Gynecology, St. Vincent’s Hospital, College of Medicine, The Catholic University of Korea, 93 Jungbudaero, Paldal-Gu, Suwon, 442-723 Gyeonggi-Do Korea

**Keywords:** Medical research, Risk factors

## Abstract

Clinical implication of a single abnormal value (SAV) in the 100 g oral glucose tolerance test during pregnancy has not been established. We aimed to evaluate the risk of postpartum type 2 diabetes mellitus (T2DM) and investigate adverse pregnancy outcomes in women with SAV, using a retrospective database, from seven medical centers of Korea. Based on the Carpenter-Coustan criteria using two-step approach, pregnancy and postpartum outcomes were compared, among normoglycemic, SAV, and gestational diabetes mellitus (GDM) groups. Among 9353 women, 342 (3.66%) and 418(4.47%) women were included in SAV and GDM groups, respectively. SAV and GDM groups showed significantly higher rates of postpartum T2DM than normoglycemic group (7.60%, 14.83%, and 1.82%, respectively, p < 0.001). And SAV group showed significantly higher rates of pregnancy associated hypertension, preterm birth, and neonatal hypoglycemia and sepsis, compared to normoglycemic group (neonatal sepsis, p = 0.008; the others, p < 0.001). In multivariate analysis, postpartum T2DM was associated with SAV, GDM (with/without insulin), nulliparity, pre-pregnancy BMI, chronic hypertension, hyperlipidemia, and DM family history. A scoring model to predict postpartum T2DM within 5 years, achieved an area under the curve of 0.74. This study demonstrated that not only GDM, but also SAV is a significant risk factor for postpartum T2DM.

## Introduction

Gestational diabetes mellitus (GDM), one of the most common complications of pregnancy, is defined as carbohydrate intolerance of variable severity, with onset or first recognition during pregnancy^1^. According to International Diabetes Federation, prevalence of GDM is approximately 16.7% in 2021, and increases with the presence of risk factors such as obesity and advanced maternal age^1–3^.

In 2014, the U.S. Preventive Services Task Force recommended blood glucose testing for all pregnant women between 24 and 28 weeks of gestation^4^. The American College of Obstetrics and Gynecology (ACOG) supports a two-step approach with a 50 g glucose challenge test (GCT) followed by a diagnostic 100 g oral glucose tolerance test (OGTT). Blood sampling was performed with fasting, and 1, 2, and 3-h after loading of the glucose solution, and diagnosis of GDM was confirmed when more than two values exceeded the National Diabetes Data Group or Carpenter- and Coustan criteria^1,5^.

The adverse maternal and neonatal outcomes of GDM are well established. In the short term, it presents risks of preeclampsia, macrosomia, shoulder dystocia, and neonatal hypoglycemia increase, along with the risks of type 2 diabetes mellitus (T2DM) and cardiovascular disease^6,7^.

Increased plasma glucose levels on glucose tolerance test are associated with adverse pregnancy outcomes. According to Hypoglycemia and Adverse Pregnancy Outcome study (HAPO study), plasma glucose levels on a maternal glucose intolerance test that were lower than those diagnosed with diabetes were also associated with adverse pregnancy outcomes, such as birth weight, cord blood C-peptide levels, primary cesarean delivery and neonatal hypoglycemia^8^.

However, the clinical implication of a single abnormal value (SAV) in the 100 g OGTT has not been established, and several studies have shown conflicting pregnancy outcomes in patients with SAV in the OGTT^9–13^.

The primary objective of this study was to evaluate the risk of type 2 DM within 5 years after delivery in women with SAV in 100 g OGTT, and the secondary objective was to investigate adverse pregnancy outcomes in women with SAV in 100 g OGTT.

## Methods

### Data source and ethical considerations

This retrospective cohort study used medical records extracted from the Clinical Data Warehouse of the Catholic Medical Center Affiliated Hospital. The study was approved by the Central Institutional Review Board of the Catholic Medical Centre (XC20WIDI0103). Because this was a retrospective cohort study and because all data were anonymized, the need for informed consent was waived by the Institutional Review Board of The Catholic University of Korea. This study was conducted in accordance with the guidelines of the Declaration of Helsinki, and the rights of all patients were protected.

### Eligibility criteria and group definition

This study included data from women who delivered between January 2009 and December 2020 at seven hospitals of the Catholic University of Korea College of Medicine. We used a two-step approach to diagnose GDM between 24 + 0 and 28 + 6 weeks of gestation. Those with an abnormal value (≥ 140 [mg/dL]) in 50 g GCT were referred to undergo 100 g OGTT. The normal cutoff value of the OGTT followed Carpenter and Coustan criteria (fasting blood glucose, 1-h, 2-h, and 3-h post-glucose loading of < 95 [mg/dL], < 180 [mg/dL], < 155 [mg/dL], and < 140 [mg/dL], respectively)^1^.

**Figure 1:**
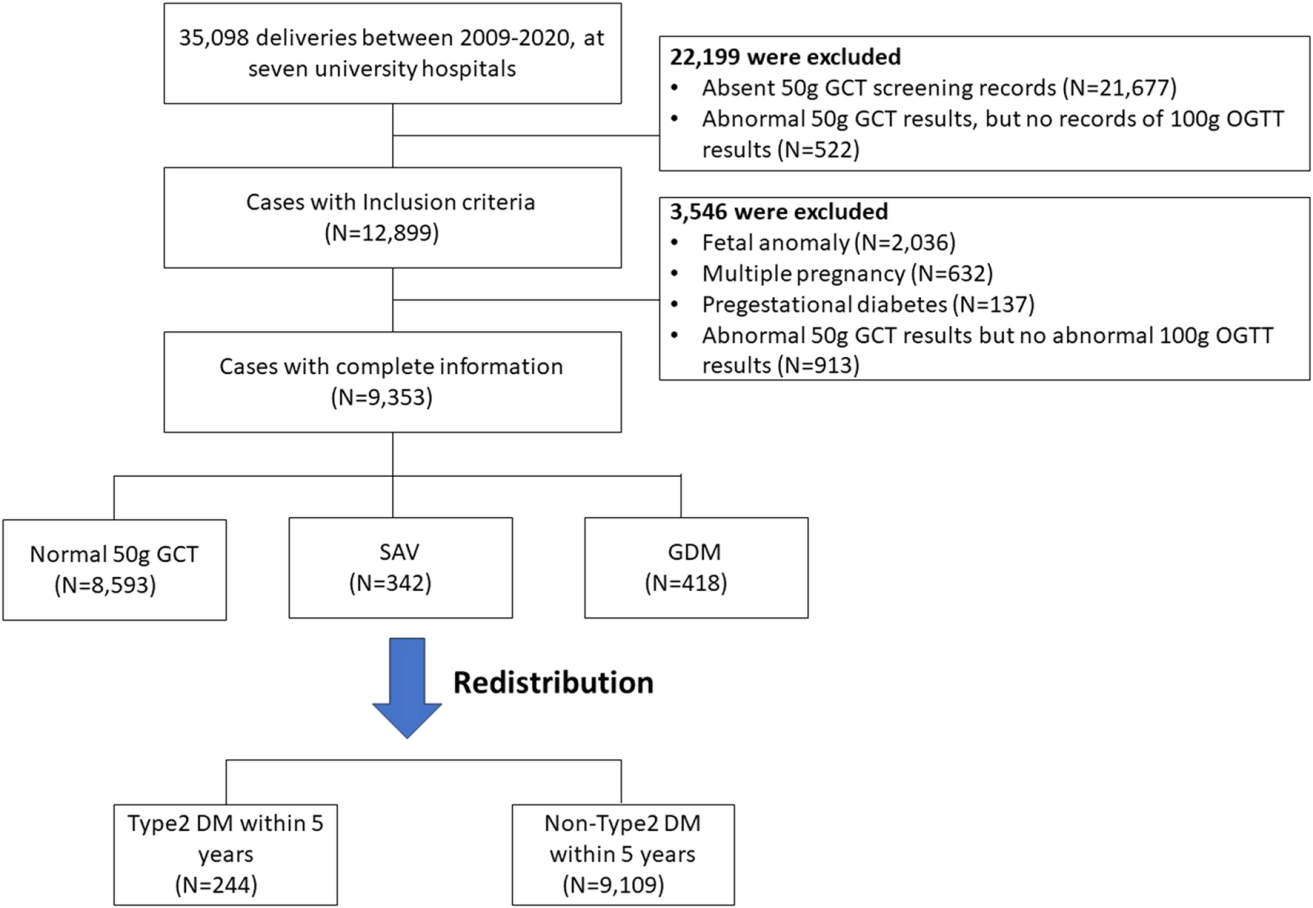
Participant flow chart of the total population. *DM* diabetes mellitus, *SAV* single abnormal value in 100 g OGTT, *OGTT* oral glucose tolerance test, *GTT* glucose tolerance test, *GDM* gestational diabetes mellitus.

We included singleton pregnancies with all medical records for GDM screening and diagnostic tests. Patients with pre-gestational diabetes, fetal anomalies, and multifetal pregnancies were excluded. Additionally, we excluded patients with a positive 50 g GCT but below the cut-off value in all criteria of the subsequent 100 g OGTT (Fig. 1).

The following three groups were compared: normal 50 g GCT (normoglycemic group), SAV in 100 g OGTT (SAV group), and two or more abnormal values in the 100 g OGTT (GDM group).

Data regarding baseline demographic characteristics, including age, parity, body mass index (BMI), conception by in vitro fertilization (IVF), 50 g GCT results, 100 g OTTT results, weight gain during pregnancy, history of chronic hypertension, chronic kidney disease, polycystic ovarian syndrome (PCO), hyperlipidemia, hypothyroidism, systemic lupus erythematosus (SLE), previous pregnancy histories of preterm birth, GDM, preeclampsia, macrosomia (baby birth weight > 4 kg), and family history of diabetes mellitus (DM), were collected. Maternal outcomes included emergency cesarean section, postpartum bleeding, pregnancy-associated hypertension (PAH), and diagnosis of postpartum diabetes (within 6 months and 5 years after delivery). Neonatal outcomes included preterm birth, gestational age at delivery, chorioamnionitis, polyhydramnios, large for gestational age (LGA), clavicle fracture, neonatal intensive care unit (NICU) admission within 48 h after birth, Apgar scores at 1 and 5 min, transient tachypnea of the newborn (TTN), respiratory distress syndrome (RDS), mechanical ventilation, use of surfactants, any grade of intraventricular hemorrhage (IVH), hypoxic ischemic encephalopathy (HIE), jaundice, hypoglycemia, and sepsis.

Preterm delivery was defined as delivery at less than 37 weeks of gestation. PAH included pre-eclampsia, superimposed pre-eclampsia, pregnancy-induced hypertension (PIH), and eclampsia. Postpartum bleeding was defined as cumulative blood loss of > 1000 mL or blood loss accompanied by signs and symptoms of hypovolemia^14^. Excessive weight gain refers to Institute of Medicine Weight Gain Recommendation for pregnancy^15^. The diagnosis of postpartum diabetes was based on the occurrence of ICD code E10-14. Additionally, we include the cases of HbA1c ≥ 6.5% or fasting blood sugar (FBS) ≥ 126 mg/dL or plasma glucose concentration ≥ 200 for 2 h after a 75 g OGTT within 5 years of delivery. The Korean Diabetes Association recommends that women with a history of gestational diabetes receive an oral glucose tolerance test 4 to 12 weeks after delivery^16^. Afterwards, these women were recommended to get screening tests for type 2 DM every year for the rest of your life, including HbA1c or FBS or OGTT. Chorioamnionitis was defined based on International Classification of Disease (ICD) code O411 or suspicious findings such as intrapartum fever, maternal leukocytosis, purulent cervical discharge, and fetal tachycardia^17^. Polyhydramnios was defined based on ICD code O40 or sonographic values exceeding an amniotic fluid index of 24 cm or a single deep pocket of 8 cm^18^. LGA was defined as cases with ICD code P08 or specified as LGA in the medical record refers to birth weight above the 90^th^ percentile for gestational age^19^. Neonatal hypoglycemia was defined based on ICD code P70 or E16 or blood sugar level < 40 mg/dL during the first 4 h of life or < 45 mg/dL during the first 4 to 24 h of life following the American academy of Pediatrics (AAP) guidelines^20^. We defined a composite neonatal outcome as one or more NICU admissions, RDS, TTN, mechanical ventilation, HIE, IVH, jaundice, hypoglycemia, and sepsis.

Furthermore, the women involved in this study were redistributed into two groups: (1) undiagnosed with diabetes within 5 years after delivery (control group), and (2) diagnosed with diabetes within 5 years after delivery (postpartum T2DM group).

Possible risk factors for postpartum T2DM were analyzed using univariate and multivariate regression analyses. For a more accurate analysis, GDM was divided into with or without insulin treatment during pregnancy.

### Statistical analysis

Baseline characteristics and pregnancy outcomes were compared between the groups using the chi-square or Fisher’s exact tests for categorical variables and t-tests for continuous variables. We conducted a post-hoc analysis using the Bonferroni method for multiple subgroup comparisons. The significance level for all statistical tests was set at p < 0.05. Variables with statistical significance in the univariate analysis were subjected to multivariate stepwise logistic regression. A scoring model for estimating the risk of T2DM within 5 years of delivery was developed using independent associated factors in the multivariate analysis. Receiver operating characteristic (ROC) analysis was performed to determine the screening performance. A Kaplan Meier Curve to make the time dynamics of postpartum T2DM occurrence was also analyzed. All analyses were performed using the Statistical Analysis Software (version 9.4; SAS Institute, Inc., Cary, NC, USA). Assistance with statistical analysis was provided by biostatisticians (YounJu Lee and Minjoo Lee) employed by contract research organization, Medical Excellence Inc., Seoul, Republic of Korea.

## Results

### Baseline characteristics according to GDM screening results

This study initially included 35,098 patient deliveries between 2009 and 2020 at the seven university hospitals. After applying the exclusion criteria, 9,353 cases were included in the study (Fig. 1). Of these, 8,593 (91.87%) cases were in the normoglycemic group, and 342 (3.66%) and 418 (4.47%) cases were in the SAV and GDM groups, respectively.

The baseline characteristics of the participants are shown in Table 1. Compared with the normoglycemic group, the SAV and GDM groups showed significantly older maternal age and higher BMI (p < 0.001). Pregnancy by IVF, the percentage of women with BMI ≥ 25 kg/m^2^ before pregnancy, BMI ≥ 30 kg/m^2^ at delivery, and chronic hypertension were also significantly higher in the SAV and GDM groups than in the normoglycemic group (p < 0.001). But weight gain (kg) during pregnancy was significantly higher in normoglycemic group than SAV and GDM groups (p < 0.001). In parous women, previous GDM history was significantly higher in the SAV and GDM groups than in the normoglycemic group (p < 0.001); however, previous histories of PAH and macrosomia were significantly higher in the GDM group than in the normoglycemic group but not in the SAV group (previous history of PAH: normoglycemic vs. GDM, p < 0.001, normoglycemic vs. SAV, p = 0.184; previous macrosomia: normoglycemic vs. GDM, p = 0.031, normoglycemic vs. SAV, p = 1.0). There was a significant difference in family history of DM and 50 g GCT results among the three groups (p < 0.001).

**Table 1:** Baseline characteristics of patients.

	Normoglycemic group (n = 8593)	SAV group (n = 342)	GDM (n = 418)	p-value*	p-value^#^	p-value^$^
Before pregnancy						
Age, mean (SD)	32.94 (4.03)	34.26 (3.97)	34.81 (4.39)	< 0.001	< 0.001	< 0.001
Nulliparity, n (%)	4778 (55.60)	192 (56.14)	218 (52.15)	0.371		
IVF, n (%)	255 (2.97)	21 (6.14)	25 (5.98)	< 0.001	0.003	0.002
BMI (kg/m^2^) before pregnancy						
Mean (SD)	21.10 (3.03)	22.28 (3.96)	23.46 (4.28)	< 0.001	< 0.001	< 0.001
< 25 kg/m^2^, n (%)	7726 (90.06)	276 (80.94)	286 (68.42)	< 0.001	< 0.001	< 0.001
≥ 25 kg/m^2^, < 30 kg/m^2^, n (%)	710 (8.28)	45 (13.20)	97 (23.21)			
≥ 30 kg/m^2^, n (%)	143 (1.67)	20 (5.87)	35 (8.37)			
Preexisting diseases						
Chronic hypertension, n (%)	356 (4.14)	27 (7.89)	47 (11.24)	< 0.001	0.002	< 0.001
CKD, n (%)	27 (0.31)	0 (0.00)	2 (0.48)	0.472		
PCO, n (%)	326 (3.79)	8 (2.34)	14 (3.35)	0.348		
Hyperlipidemia, n (%)	218 (2.54)	9 (2.63)	23 (5.50)	0.001	1.0	< 0.001
Hypothyroidism, n (%)	552 (6.42)	32 (9.36)	26 (6.22)	0.095		
SLE, n (%)	105 (1.22)	3 (0.88)	2 (0.48)	0.445		
Previous preterm delivery history, n (%)	493 (5.74)	30 (8.77)	39 (9.33)	0.046	0.130	0.025
Previous GDM history, n (%)	68 (0.79)	11 (3.22)	17 (4.07)	< 0.001	< 0.001	< 0.001
Previous PAH history^†^, n (%)	172 (2.00)	13 (3.80)	22 (5.26)	< 0.001	0.184	< 0.001
Previous macrosomia^††^, n (%)	134 (1.62)	8 (2.42)	14 (3.45)	0.039	1.0	0.031
Family history of DM, n (%)	894 (10.40)	51 (14.91)	94 (22.49)	< 0.001	0.024	< 0.001
During pregnancy						
BMI (kg/m^2^) at delivery						
Mean (SD)	26.12 (3.35)	27.03 (4.19)	27.28 (4.20)	< 0.001	0.002	< 0.001
< 25 kg/m^2^, n (%)	3510 (40.90)	122 (35.67)	134 (32.06)	< 0.001	< 0.001	< 0.001
≥ 25 kg/m^2^, < 30 kg/m^2^, n (%)	4067 (47.39)	153 (44.74)	189 (45.22)			
≥ 30 kg/m^2^, n (%)	1005 (11.71)	67 (19.59)	95 (22.73)			
Weight gain (kg), mean (SD)	13.11 (4.62)	12.30 (4.95)	9.86 (5.15)	< 0.001	< 0.001	< 0.001
Excessive weight gain^†††^, n (%)	1956 (22.80)	77 (22.58)	65 (15.55)	< 0.001	1.0	0.002
Gestational age at 50 g GCT, mean (SD)	25.14 (1.13)	25.11 (1.19)	24.94 (1.24)	0.013	1.0	0.049
50 g GCT results						
Mean (SD)	110.30 (16.72)	157.84 (14.54)	167.45 (23.92)	< 0.001	< 0.001	< 0.001
< 140	8593 (100.0)	0 (0.00)	0 (0.00)	< 0.001	< 0.001	< 0.001
≥ 140, < 160	0 (0.00)	218 (63.74)	198 (47.37)			
≥ 160	0 (0.00)	124 (36.26)	220 (52.63)			
Gestational age at 100 g OGTT, mean (SD)	26.04 (1.22)	25.91 (1.13)	25.97 (1.27)	0.211		
100 g OGTT results, mean (SD)						
Fasting	83.95 (7.95)	88.01 (18.06)	90.44 (12.44)	< 0.001	0.807	0.006
After 1 h	146.99 (25.11)	162.85 (23.81)	189.44 (28.42)	< 0.001	0.001	< 0.001
After 2 h	136.42 (24.60)	146.24 (21.83)	178.42 (27.17)	< 0.001	0.122	< 0.001
After 3 h	115.51 (26.11)	121.87 (21.92)	143.43 (30.83)	< 0.001	0.642	< 0.001

Values are expressed as means (standard deviation, SD), or n (%).

*SAV* single abnormal value in 100 g OGTT, *OGTT* oral glucose tolerance test, *GDM* gestational diabetes mellitus, *IVF* in vitro fertilization, *BMI* body mass index, *CKD* chronic kidney disease, *PCO* polycystic ovarian syndrome, *SLE* systemic lupus erythematosus, *PAH* pregnancy-associated hypertension, *DM* diabetes mellitus, *GCT* glucose challenge test, *OGTT* oral glucose tolerance test.

*p-value: normoglycemic vs SAV vs GDM.

^#^Post-hoc p-value using Bonferroni methods: normoglycemic vs SAV.

^$^Post-hoc p-value using Bonferroni methods: normoglycemic vs GDM.

^†^PAH: corresponding to one of preeclampsia, superimposed preeclampsia, pregnancy-induced hypertension, and eclampsia.

^††^macrosomia: corresponding to baby birth w eight above 4 kg.

^†††^Excessive weight gain: refers to Institute of Medicine Weight Gain Recommendation for pregnancy.

### Pregnancy outcomes according to GDM screening results

The data on adverse pregnancy and neonatal outcomes are presented in Table 2. Compared with the normoglycemic group, the SAV and GDM groups showed significantly higher rates of preterm birth, PAH, neonatal hypoglycemia, and NICU admissions (preterm birth, p < 0001; PAH, p < 0.001; neonatal hypoglycemia, p < 0.001; NICU admissions, p = 0.023). There were significantly higher rates of RDS, use of surfactants, jaundice, and neonatal composite outcomes in the GDM group than in the normoglycemic group (RDS, p = 0.01; use of surfactant, p = 0.003; jaundice, p = 0.001; neonatal composite outcome, p = 0.004). However, there was a significantly higher rate of sepsis in the SAV group than in the normoglycemic group, but not in the GDM group (normoglycemic vs. SAV, p = 0.007; normoglycemic vs. GDM, p = 1.0).

**Table 2:** Maternal and neonatal outcomes according to OGTT results.

	Normoglycemic group (n = 8,593)	SAV group (n = 342)	GDM (n = 418)	p-value*	p-value^#^	p-value^$^
Maternal outcomes						
Emergency cesarean section, n (%)	836 (24.57)	35 (22.29)	55 (23.50)	0.765		
Postpartum bleeding, n (%)	399 (4.64)	13 (3.80)	26 (6.22)	0.242		
PAH^†^, n (%)	313 (3.64)	28 (8.19)	43 (10.29)	< 0.001	< 0.001	< 0.001
Diabetes diagnosis within 6 months after delivery, n (%)	1 (0.01)	0 (0.00)	4 (0.96)	< 0.001	1.0	< 0.001
Diabetes diagnosis within 5 years after delivery, n (%)	156 (1.82)	26 (7.60)	62 (14.83)	< 0.001	< 0.001	< 0.001
Neonatal outcomes						
Preterm birth, n (%)	1676 (19.50)	94 (27.49)	114 (27.27)	< 0.001	0.001	< 0.001
Gestational age at delivery (weeks)						
Mean (SD)	38.76 (1.57)	38.39 (2.19)	38.21 (1.80)	< 0.001	0.007	< 0.001
< 24 + 0, n (%)	0 (0.00)	2 (0.58)	0 (0.00)	< 0.001	< 0.001	0.014
≥ 24 + 0, < 28 + 0, n (%)	18 (0.21)	1 (0.29)	3 (0.72)			
≥ 28 + 0, < 34 + 0, n (%)	102 (1.19)	7 (2.05)	6 (1.44)			
≥ 34 + 0, < 37 + 0, n (%)	444 (5.17)	27 (7.89)	35 (8.37)			
≥ 37 + 0, n (%)	8029 (93.44)	305 (89.18)	374 (89.47)			
Chorioamnionitis, n (%)	13 (0.15)	2 (0.58)	2 (0.48)	0.054		
Polyhydramnios, n (%)	27 (0.31)	2 (0.58)	4 (0.96)	0.058		
LGA, n (%)	194 (2.26)	9 (2.63)	14 (3.35)	0.325		
Clavicle fracture, n (%)	23 (0.27)	2 (0.58)	1 (0.24)	0.330		
NICU admission, n (%)	1068 (12.43)	57 (16.67)	63 (15.07)	0.023	0.062	0.334
Apgar score < 5 in 1 min, n (%)	198 (2.31)	14 (4.09)	11 (2.63)	0.099		
Apgar score < 5 in 5 min, n (%)	53 (0.62)	4 (1.17)	2 (0.48)	0.416		
RDS, n (%)	126 (1.47)	5 (1.46)	14 (3.35)	0.01	1.0	0.007
Mechanical ventilation, n (%)	464 (5.40)	25 (7.31)	28 (6.70)	0.178		
Use of surfactant, n (%)	71 (0.83)	4 (1.17)	11 (2.63)	0.003	1.0	0.004
IVH, n (%)	114 (1.33)	5 (1.46)	2 (0.48)	0.313		
HIE, n (%)	226 (2.63)	13 (3.80)	11 (2.63)	0.42		
Jaundice, n (%)	1362 (15.85)	61 (17.84)	95 (22.73)	0.001	0.975	< 0.001
Hypoglycemia, n (%)	17 (0.20)	7 (2.05)	35 (8.37)	< 0.001	< 0.001	< 0.001
Sepsis, n (%)	187 (2.18)	16 (4.68)	8 (1.91)	0.008	0.007	1.0
Composite outcome^††^, n (%)	1977 (23.01)	92 (26.90)	122 (29.19)	0.004	0.282	0.011

Values are expressed as means (standard deviation, SD), or n (%).

*SAV* single abnormal value in 100 g OGTT, *OGTT* oral glucose tolerance test, *GDM* gestational diabetes mellitus, *PAH* pregnancy-associated hypertension, *LGA* large for gestational age, *NICU* neonatal intensive care unit, *RDS* respiratory distress syndrome, *IVH* intraventricular hemorrhage, *HIE* hypoxic ischemic encephalopathy.

*p-value: normoglycemic vs SAV vs GDM.

^#^Post-hoc p-value using Bonferroni methods: normoglycemic vs SAV.

^$^Post-hoc p-value using Bonferroni methods: normoglycemic vs GDM.

^†^PAH: corresponding to one of preeclampsia, superimposed preeclampsia, pregnancy-induced hypertension, and eclampsia.

^††^Composite outcome: corresponding to one or more of NICU admission, RDS, TTN, mechanical ventilation, HIE, IVH, Jaundice, hypoglycemia, sepsis.

Regarding postpartum prognosis, there was a significant difference among the three groups in the diagnosis of T2DM within 6 months and 5 years after delivery (p < 0.001). In the post hoc test, the diagnosis of diabetes within 6 months after delivery significantly differed between the normoglycemic and GDM groups (normoglycemic vs. GDM, p < 0.001; normoglycemic vs. SAV, p = 1.0). Additionally, the diagnosis of diabetes within 5 years after delivery showed significant differences among the three groups (p < 0.001).

### Maternal characteristics according to the diagnosis of T2DM within 5 years after delivery

When the postpartum T2DM group was compared with the control group, women in the postpartum T2DM group had a significantly higher rate of SAV and GDM (with or without insulin treatment) following GCT were significantly higher in the postpartum T2DM group than in the control group (p < 0.001) (Table 3). Women in the postpartum T2DM group also had significantly higher rates of nulliparity, obesity, chronic hypertension, PCO, hyperlipidemia, hypothyroidism, previous GDM history, previous preeclampsia history, PAH in the current pregnancy, and family history of DM, compared to women in the control group. But mean weight gain during pregnancy was significantly higher in control group than postpartum T2DM group (p < 0.001) and there was no significant difference in the proportion of women with excessive weight gain between control and postpartum T2DM groups.

**Table 3:** Maternal characteristics according to diagnosis of postpartum T2DM.

	Control group (n = 9,109)	Postpartum T2DM (n = 244)	p-value
Age			
Mean (SD)	33.06 (4.07)	33.43 (4.18)	0.244
≥ 35 years, n (%)	3214 (35.28)	96 (39.34)	0.191
< 35 years, n (%)	5895 (64.72)	148 (60.66)	
Nulliparity, n (%)	5033 (55.25)	155 (63.52)	0.01
IVF, n (%)	290 (3.18)	11 (4.51)	0.247
BMI before pregnancy, kg/m^2^			
Mean (SD)	21.21 (3.13)	22.79 (4.34)	< 0.001
< 25 kg/m^2^, n (%)	8108 (89.16)	180 (73.77)	< 0.001
≥ 25 kg/m^2^, < 30 kg/m^2^, n (%)	805 (8.85)	47 (19.26)	
≥ 30 kg/m^2^, n (%)	181 (1.99)	17 (6.97)	
BMI at delivery, kg/m^2^			
Mean (SD)	26.17 (3.40)	27.36 (4.57)	0.001
< 25 kg/m^2^, n (%)	3678 (40.43)	88 (36.07)	< 0.001
≥ 25 kg/m^2^, < 30 kg/m^2^, n (%)	4314 (47.42)	95 (38.93)	
≥ 30 kg/m^2^, n (%)	1106 (12.16)	61 (25.00)	
Weight gain (kg) during pregnancy, mean (SD)	12.96 (4.68)	11.85 (5.40)	< 0.001
Excessive weight gain during pregnancy^†^, n (%)	2048 (22.52)	50 (20.49)	0.454
PAH^††^, n (%)	366 (4.02)	18 (7.38)	0.009
50 g GCT results, mean (SD)	114.04 (21.71)	135.32 (33.95)	< 0.001
100 g OGTT results, mean (SD)			
Fasting	88.42 (13.96)	94.57 (21.19)	0.0005
After 1 h	175.24 (29.12)	185.99 (35.27)	0.0016
After 2 h	161.62 (29.16)	174.95 (33.54)	0.001
After 3 h	133.67 (29.27)	148.24 (34.19)	< 0.001
GDM screening results			< 0.001
Normoglycemic^†††^, n (%)	8437 (92.62)	156 (63.93)	
SAV, n (%)	316 (3.47)	26 (10.66)	
GDM- IT, n (%)	108 (1.19)	37 (15.6)	
GDM + IT, n (%)	248 (2.72)	25 (10.25)	
Preexisting diseases			
Chronic hypertension, n (%)	403 (4.42)	27 (11.07)	< 0.001
PCO, n (%)	332 (3.64)	16 (6.56)	0.018
Hyperlipidemia, n (%)	233 (2.56)	17 (6.97)	< 0.001
Hypothyroidism, n (%)	586 (6.43)	24 (9.84)	0.034
RA, n (%)	343 (3.77)	12 (4.92)	0.353
SLE, n (%)	106 (1.16)	4 (1.64)	0.537
Previous GDM history, n (%)	89 (0.98)	7 (2.87)	< 0.001
Previous PAH^†^ history, n (%)	198 (2.17)	9 (3.69)	0.005
Family history of DM, n (%)	984 (10.80)	55 (22.54)	< 0.001

Values are expressed as means (standard deviation, SD), or n (%).

*T2DM* type 2 diabetes mellitus, *BMI* body mass index, *PCO* polycystic ovarian syndrome, *RA* rheumatic arthritis, *SLE* systemic lupus erythematosus, *DM* diabetes mellitus, *IVF* in vitro fertilization, *PAH* pregnancy-associated hypertension, *GCT* glucose challenge test, *GDM* gestational diabetes mellitus, *SAV* single abnormal value in 100 g OGTT, *OGTT* oral glucose tolerance test, *GDM-IT* GDM not requiring insulin treatment, *GDM* + *IT* GDM requiring insulin treatment.

^†^Excessive weight gain refers to Institute of Medicine Weight Gain Recommendation for pregnancy.

^††^PAH: corresponding to one of preeclampsia, superimposed preeclampsia, pregnancy-induced hypertension, and eclampsia.

^†††^Normoglycemic: corresponding to normal 50 g GCT results.

### Univariate and stepwise multivariate logistic regression analysis predicting risk for postpartum T2DM

**Figure 2:**
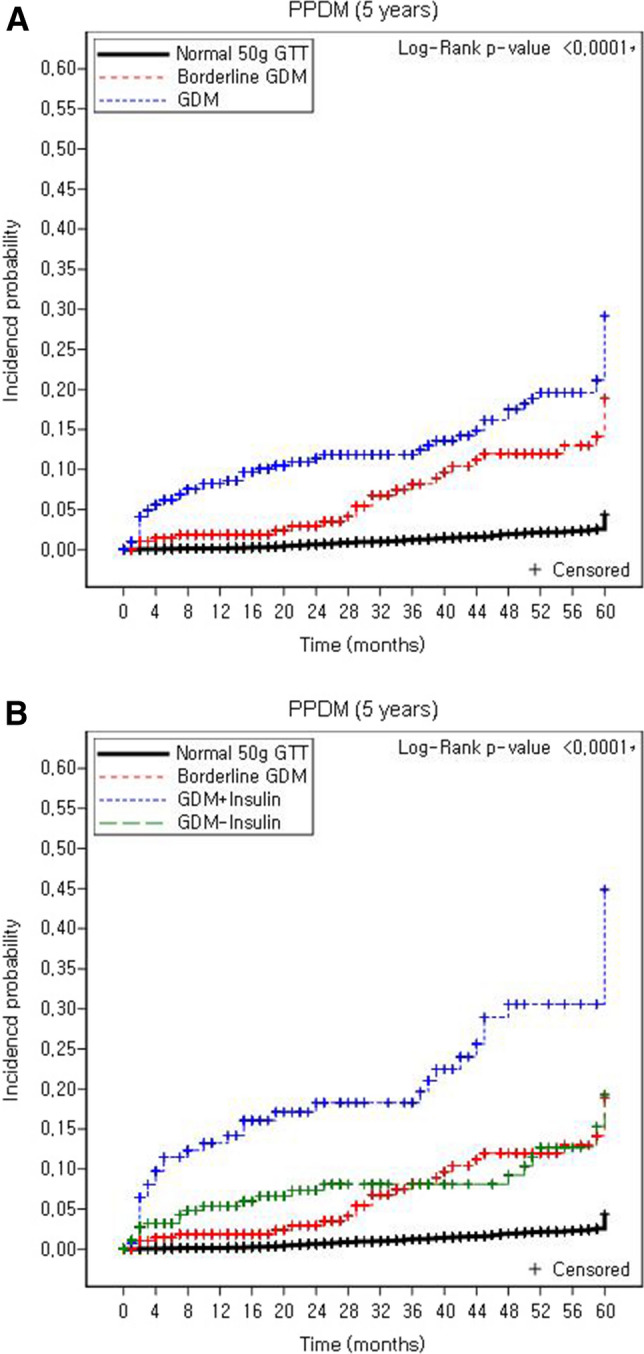
Kaplan–Meier curves for time to T2DM diagnosis according to GDM screening results. (**A**) Comparison between normoglycemic, SAV, GDM, (**B**) Comparison between normoglycemic, SAV, GDM not requiring insulin, GDM requiring insulin.

In the univariate analysis, ORs of SAV, GDM (with or without insulin treatment), nulliparity, BMI before pregnancy ≥ 25 kg/m^2^, < 30 kg/m^2^, BMI before pregnancy ≥ 30 kg/m^2^, BMI at delivery ≥ 30 kg/m^2^, chronic hypertension, PCO, hyperlipidemia, hypothyroidism, previous GDM history, previous PAH history, family history of DM and pregnancy complication of PAH were significantly increased in the postpartum T2DM group (Table 4). Stepwise multivariate logistic regression analysis was performed using variables that were significantly different between the postpartum T2DM and control groups in the univariate analysis. In the multivariate logistic regression analysis, SAV (odds ratio [OR] 3.95, 95% confidence interval [CI] 2.55–6.11), GDM not requiring insulin treatment (OR 4.30, 95% CI 2.73–6.77), GDM requiring insulin treatment (OR 13.21, 95% CI 8.58–20.34), nulliparity (OR 1.58, 95% CI 1.21–2.08), BMI before pregnancy ≥ 25 kg/m^2^, < 30 kg/m^2^ (OR 1.81, 95% CI 1.27–2.58), BMI before pregnancy ≥ 30 kg/m^2^ (OR 1.90, 95% CI 1.06–3.43), chronic hypertension (OR 1.82, 95% CI 1.16–2.86), hyperlipidemia (OR 1.90, 95% CI 1.08–3.34) and family history of DM (OR 1.93, 95% CI 1.40–2.68) were significantly associated with postpartum T2DM (Table 4). Kaplan–Meier curves showed that cumulative incidence of T2DM diagnosis within 5 years after birth was significantly higher in the GDM and SAV group as presented in Fig. 2.

**Table 4:** Odd ratios for risk factors of postpartum T2DM using univariate and multivariate stepwise logistic regression analysis.

	Univariate analysis	p-value	Multivariate analysis	p-value
	OR (95% CI)		OR (95% CI)	
SAV (ref. normoglycemic group)	4.45 (2.89, 6.84)	< 0.001	3.95 (2.55, 6.11)	< 0.001
GDM-IT (ref. normoglycemic group)	5.45 (3.51, 8.47)	< 0.001	4.30 (2.73, 6.77)	< 0.001
GDM + IT (ref. normoglycemic group)	18.53 (12.35, 27.80)	< 0.001	13.21 (8.58, 20.34)	< 0.001
Age ≥ 35 years (ref. < 35 years)	1.19 (0.92, 1.54)	0.191		
Nulliparity (ref. No)	1.41 (1.08, 1.84)	0.011	1.58 (1.21, 2.08)	0.001
BMI before pregnancy ≥ 25 kg/m^2^, < 30 kg/m^2^ (ref. < 25 kg/m^2^)	2.63 (1.89, 3.66)	< 0.001	1.81 (1.27, 2.58)	0.001
BMI before pregnancy ≥ 30 kg/m^2^ (ref. < 25 kg/m^2^)	4.23 (2.52, 7.11)	< 0.001	1.90 (1.06, 3.43)	0.033
BMI at delivery ≥ 25 kg/m^2^, < 30 kg/m^2^ (ref. < 25 kg/m^2^)	0.92 (0.69, 1.23)	0.579		
BMI at delivery ≥ 30 kg/m^2^ (ref. < 25 kg/m^2^)	2.31 (1.65, 3.22)	< 0.001		
Excessive weight gain during pregnancy^†^ (ref. No)	0.89 (0.65, 1.22)	0.45		
Chronic hypertension (ref. No)	2.69 (1.78, 4.06)	< 0.001	1.82 (1.16, 2.86)	0.01
PCO (ref. No)	1.86 (1.11, 3.12)	0.02		
Hyperlipidemia (ref. No)	2.85 (1.71, 4.75)	< 0.001	1.90 (1.08, 3.34)	0.026
Hypothyroidism (ref. No)	1.59 (1.03, 2.44)	0.035		
Previous GDM history	3.83 (1.72, 8.51)	0.001		
Previous PAH^††^ history (ref. No)	2.20 (1.09, 4.45)	0.028		
Family history of DM (ref. No)	2.40 (1.77, 3.27)	< 0.001	1.93 (1.40, 2.68)	< 0.001
Pregnancy complication of PAH^†^ (ref. No)	1.90 (1.16, 3.11)	0.010		

*CI* confidence interval, *OR* odds ratio, *T2DM* type 2 diabetes mellitus, *SAV* single abnormal value in 100 g OGTT, *OGTT* oral glucose tolerance test, *GDM* gestational diabetes mellitus, *BMI* body mass index, *PCO* polycystic ovarian syndrome, *DM* diabetes mellitus, *PAH* pregnancy-associated hypertension, *GDM-IT* GDM not requiring insulin treatment, *GDM* + *IT* GDM requiring insulin treatment.

^†^Excessive weight gain: refers to Institute of Medicine Weight Gain Recommendation for pregnancy.

^††^PAH: corresponding to one of preeclampsia, superimposed preeclampsia, pregnancy-induced hypertension, and eclampsia.

### Stratified risk score for predicting Postpartum T2DM

**Figure 3:**
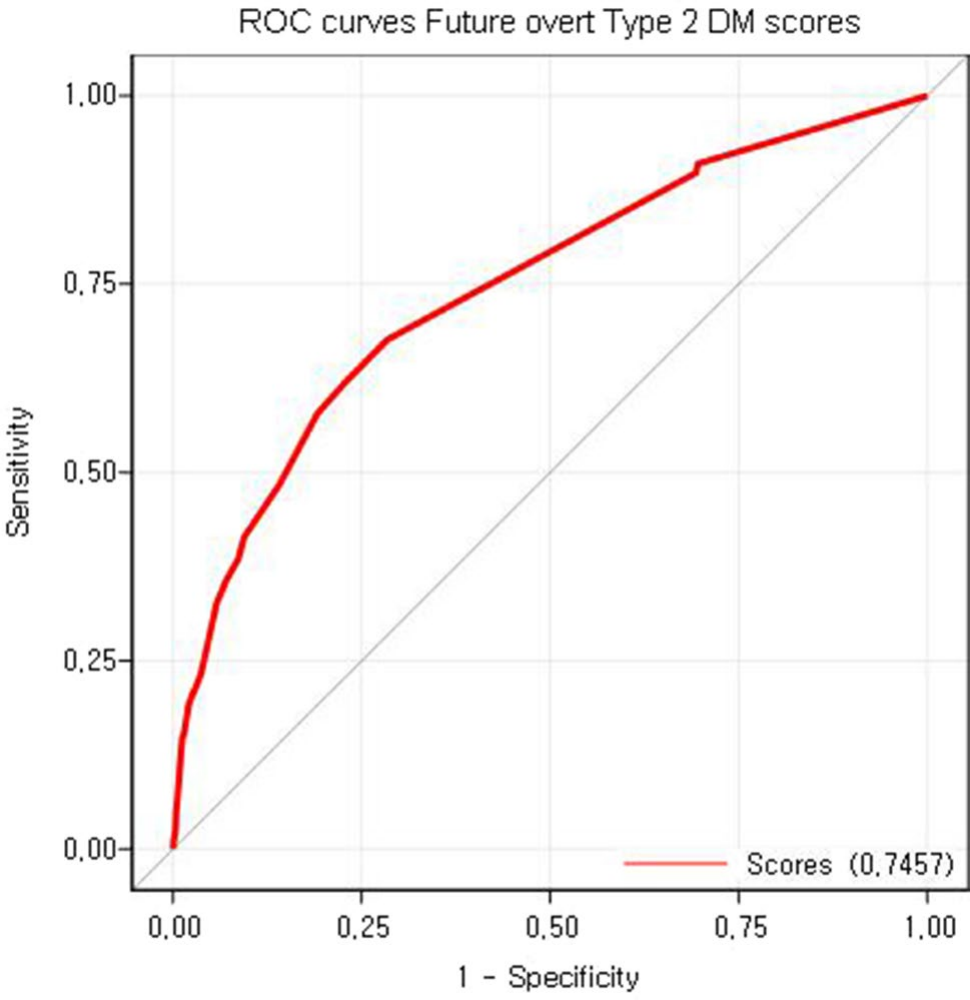
Receiver operating characteristic curve of the scoring model for postpartum type 2 diabetes mellitus within 5 years post-delivery.

We developed a statistical scoring model using independent risk factors to estimate the individual risks of postpartum T2DM (Table 5). In ROC analysis, the scoring model achieved an area under the ROC curve (AUC) of 0.746 (Fig. 3).

**Table 5:** Risk scores for predicting postpartum -T2DM within 5 years after birth.

Risk factor	Categories	Points
BMI before pregnancy	< 25 kg/m^2^	0
	≥ 25 kg/m^2^, < 30 kg/m^2^	5
	≥ 30 kg/m^2^	5
Family history of DM	Yes	6
	No	0
Glucose screening test results	Normoglycemic^†^	0
	SAV	12
	GDM − IT	12
	GDM + IT	22
Nulliparity	Yes	4
	No	0
Chronic hypertension	Yes	5
	No	0
Hyperlipidemia	Yes	5
	No	0

Point total	Estimate of risk (%)	
0	1.11	
1	1.24	
2	1.40	
3	1.57	
4	1.76	
5	1.98	
6	2.23	
7	2.50	
8	2.80	
9	3.15	
10	3.53	
11	3.95	
12	4.43	
13	4.96	
14	5.55	
15	6.20	
16	6.93	
17	7.73	
18	8.62	
19	9.60	
20	10.68	
21	11.86	
22	13.16	
23	14.57	
24	16.11	
25	17.78	
26	19.58	
27	21.51	
28	23.58	
29	25.78	
30	28.11	
31	30.56	
32	33.13	
33	35.81	
34	38.58	
35	41.42	
36	44.32	
37	47.26	
38	50.22	
39	53.18	
40	56.11	
41	59.01	
42	61.84	
43	64.59	
44	67.25	
45	69.81	
46	72.24	
47	74.56	

*BMI* body mass index, *DM* diabetes mellitus, *SAV* single abnormal value in 100 g OGTT, *OGTT* oral glucose tolerance test, *GDM* gestational diabetes mellitus, *GDM-IT* GDM not requiring insulin treatment, *GDM* + *IT* GDM requiring insulin treatment.

^†^Normoglycemic: corresponding to normal 50 g GCT results.

## Discussion

### Principal findings

This study demonstrated that SAV was a significant risk factor for postpartum T2DM. In addition, the Kaplan Meier curve showed similar occurrence of T2DM between SAV and GDM without insulin groups, within 5 years after birth. Women with SAV had clinical characteristics similar to those with GDM, including older maternal age, higher BMI, conception by IVF, chronic hypertension, previous GDM history, and family history of DM. We also found a higher incidence of adverse pregnancy outcomes, including preterm birth, neonatal hypoglycemia, neonatal sepsis, NICU admissions, and PAH in the SAV group than in the normoglycemic group.

### Clinical implications

A positive correlation between maternal glucose levels on glucose tolerance test and adverse pregnancy outcomes is well known^8^. Simmon et al. studied 802 subjects and found that starting GDM treatment at an early gestational age reduced adverse neonatal outcomes^21^. As the prognosis of pregnancy varies depending on glycemic control, it is important to diagnose and manage GDM accurately.

GDM diagnosis can be accomplished with either of two strategies: (1) “One step” 75 g OGTT or (2) a “Two-step” approach with a 50 g GCT followed by 100 g OGTT for screening the positive group^5^. The number of patients diagnosed may vary depending on the application of different diagnostic criteria. Previous studies have reported more diagnoses of GDM using a one-step approach than using a two-step approach^22,23^. Whether there is overtreatment using a one-step approach or under diagnosis using a two-step approach is debatable.

Several studies have found that using a one-step approach increases the diagnostic rate of GDM but does not improve obstetric and neonatal outcomes^24–26^. Based on these findings, the ACOG supports a two-step approach^1^. However, those who oppose this approach claim that the GDM group with a one-step approach has a 3.4-fold higher risk of T2DM; additionally, children with GDM had a higher risk of obesity than the normoglycemic group when observed for 11 years^27,28^. The American Diabetes Association concluded that GDM diagnosis can be accomplished with either of the two strategies; however, there is still controversy regarding which method is better regarding long-term prognosis^5^.

When only SAV is considered in a two-step approach, it is called borderline GDM, impaired glucose tolerance, or mild hyperglycemia^29–31^. Previous studies have suggested that SAV and GDM occur via the same mechanism caused by increased insulin resistance during pregnancy^32–36^. A meta-analysis of 25 studies demonstrated that patients with SAV on a 100 g OGTT had increased LGA, neonatal hypoglycemia, and cesarean delivery^37^. A randomized controlled trial reported similar perinatal outcomes in SAV and untreated mild GDM^38^. In prospective study, pregnancy outcomes improved when women with SAV were managed similarly to those with GDM^39^. As a long-term outcome, several studies concluded that SAV diagnosed during pregnancy increases future abnormal glucose tolerance similar to GDM^32,35,40,41^. Moreover, previous study found that SAV is independent risks factor of T2DM after 5 years of delivery^41^.

Our study also demonstrated that not only GDM, but also SAV during pregnancy were significant risk factor for postpartum T2DM. We subdivided the GDM group according to whether or not they were treated with insulin during pregnancy. There was similar risk of postpartum T2DM in SAV group, compared to GDM without insulin treatment. This study suggests postpartum life-style modification in women with SAV, although future studies may be needed whether postpartum monitoring or preventive education in women with SAV is useful for their long-term health.

### Strengths and limitations

Our data were obtained from seven centers in different regions of Korea, which have a same diagnostic protocol for GDM. In this study, SAV showed a comparable risk of postpartum T2DM to women with GDM who did not require insulin during pregnancy. Using independent risk factors from this study, we developed a scoring system that can be applied to individual patients. In this study, the average BMI and the proportion of obesity tended to be low. Although the obese population is increasing in Korea, the obesity rate is still lower than in the West. However, it is known that Asian women have moderate risk of GDM, despite having a relatively lower BMI compared to other ethnic groups^42^. Therefore, this study may be more meaningful as a risk model for this population, although future validation studies are required for clinical application,

Our study is not free for limitations because of retrospective nature. First, there were missing data for those who did not receive a 75 g OGTT test after delivery. Second, cases with abnormal 50 g GCT but no abnormal OGTT results or 100 g OGTT were excluded. This group was clearly judged to be low risk in the confirmatory test. Therefore, there was no need to compare the risk with the group that was already being managed as a low-risk group because the 50 g test was negative. Previous study comparing between a 50 g negative group and a 50 g positive but negative in all results of 100 g OGTT showed that there was no difference in pregnancy outcomes between the two groups^43^. Lastly, incidence of GDM was relatively low in this study. In Korea, GDM prevalence has been increased from 7.5% in 2009 to 18.2% in by 2021^42^. The low prevalence of GDM in our study was influenced by the inclusion of historical data from 2009. This also has the impact of excluding patients with GDM who have already been diagnosed and transferred from another hospital. To ensure the accuracy of the study, only patients who receive all tests for GDM in our hospitals were included.

To overcome the limitation of retrospective data, the validation cohort is currently being enrolled. We will use these prospective data to demonstrate the clinical significance of SAV and validate our scoring system and apply it to individual patients.

## Conclusion

In clinical practice, women with SAV should not be neglected, particularly regarding the long-term risk of postpartum T2DM. Although more prospective studies are needed, the individual risk-scoring model, including SAV in this study, may be helpful for long-term lifestyle modifications to decrease the risk of postpartum T2DM.

## Data Availability

The datasets generated and/or analysed during the current study are not publicly available due to the sensitivity of the data but are available from the corresponding author upon reasonable request.

## References

[1] Bulletins Obstetrics, C (2018). ACOG practice bulletin, NO. 190: Gestational diabetes mellitus. Obstet. Gynecol..

[2] International Diabetes Federation. *IDF Diabetes Atlas*. 10th edition, https://diabetesatlas.org/idfawp/resource-files/2021/07/IDF_Atlas_10th_Edition_2021.pdf

[3] Greenberg VR (2022). Perinatal outcomes in obese women with one abnormal value on 3-hour oral glucose tolerance test. Am. J. Perinatal..

[4] Moyer VA (2014). Screening for gestational diabetes mellitus: U.S. Preventive Services Task Force recommendation statement. Ann. Intern. Med..

[5] American Diabetes Association (2024). Diagnosis and classification of diabetes: Standards of care in diabetes-2024. Diabetes Care.

[6] American Diabetes Association (2024). Management of Diabetes in pregnancy: Standards of Care in Diabetes-2024. Diabetes Care.

[7] Kramer CK, Campbell S, Retnakaran R (2019). Gestational diabetes and the risk of cardiovascular disease in women: a systematic review and meta-analysis. Diabetologia.

[8] HAPO Study Cooperative Research Group (2024). Hyperglycemia and adverse pregnancy outcomes. N. Engl. J. Med..

[9] Langer O, Brustman L, Anyaegbunam A, Mazze R (1987). The significance of one abnormal glucose tolerance test value on adverse outcome in pregnancy. Am. J. Obstet. Gynecol..

[10] Lindsay MK, Graves W, Klein L (1989). The relationship of one abnormal glucose tolerance test value and pregnancy complication. Obstet. Gynecol..

[11] Forest JC, Masse J, Garrido-Russo M (1994). Glucose tolerance test during pregnancy: the significance of one abnormal value. Clin. Biochem..

[12] Sermer, M. *et al*. Impact of increasing carbohydrate intolerance on maternal-fetal outcome in 3637 women without gestational diabetes. The Toronto Tri-Hospital Gestational Diabetes Project. *Am. J. Obstet. Gynecol.* **173**, 146–156 (1995). 10.1016/0002-9378(95)90183-3 7631672

[13] Vambergue A (2002). Pregnancy induced hypertension in women with gestational carbohydrate intolerance: The diagest study. Eur. J. Obstet. Gynecol. Reprod. Biol..

[14] Committee on Practice Bulletins-Obstetrics (2017). ACOG practice bulletin, No.183: Postpartum Hemorrhage. Obstet. Gynecol..

[15] Rasmussen, K. *Weight Gain During Pregnancy: Reexamining the Guidelines* (Institute of Medicine (US) and National Research Council (US) Committee to Reexamine IOM Pregnancy Weight Guidelines, 2009). 20669500

[16] Korean Diabetes Association (2023). Clinical Practice Guidelines for Diabetes (8th edition).

[17] Committee Opinion No. 712: Intrapartum management of intraamniotic infection. *Obstet. Gynecol.* **130**, e95–e101 (2017). 10.1097/AOG.0000000000002236 28742677

[18] Chamberlain PF, Manning FA, Morrison I, Harman CR, Lange IR (1984). Ultrasound evaluation of amniotic fluid volume. I. The relationship of marginal and decreased amniotic fluid volumes to perinatal outcome. Am. J. Obstet. Gynecol..

[19] Lee, J. K. *et al*. Percentile distribution of birth weight according to gestational ages in Korea (2010–2012). *J. Korean Med. Sci.* **31**, 939–949 (2016). 10.3346/jkms.2016.31.6.939 PMC4853674 27247504

[20] Adamkin DK (2017). Neonatal hypoglycemia. Semin. Fetal Neonatal Med..

[21] Simmons D (2023). Treatment of gestational diabetes mellitus diagnosed early in pregnancy. N. Engl. J. Med..

[22] Hillier TA (2021). A pragmatic, randomized clinical trial of gestational diabetes screening. N. Engl. J. Med..

[23] Coustan DR, Dyer AR, Metzger BE (2021). Perinatal outcomes of two screening strategies for gestational diabetes mellitus. Obstet. Gynecol..

[24] Ghaffari N, Gonzalez JM, Rosenstein MG (2020). Does the 1-step method of gestational diabetes mellitus screening improve pregnancy outcomes?. Am. J. Obstet. Gynecol..

[25] Feldman RK, Tieu RS, Yasumura L (2016). Gestational diabetes screening: The International Association of the Diabetes and Pregnancy Study Groups compared with Carpenter-Coustan screening. Obstet. Gynecol..

[26] Pocobelli G (2018). One-step approach to identifying gestational diabetes mellitus: Association with perinatal outcomes. Obstet. Gynecol..

[27] Lowe WL (2018). Association of gestational diabetes with maternal disorders of glucose metabolism and childhood adiposity. JAMA.

[28] Lowe WL (2019). Hyperglycemia and Adverse pregnancy outcome follow-up study (HAPO FUS): Maternal gestational diabetes mellitus and childhood glucose metabolism. Diabetes Care.

[29] Naylor, C. D. *et al*. Cesarean delivery in relation to birth weight and gestational glucose tolerance: pathophysiology or practice style? Toronto Trihospital Gestational Diabetes Investigators. *JAMA* **275**, 1165–1170 (1996). 8609683

[30] Berkus MD, Langer O (1993). Glucose tolerance test: Degree of glucose abnormality correlates with neonatal outcome. Obset. Gynecol..

[31] Vambergue A (2000). Is mild gestational hyperglycemia associated with maternal and neonatal complications? The Diagest Study. Diabet. Med..

[32] Corrado F (2007). Positive association between a single abnormal glucose tolerance test value in pregnancy and subsequent abnormal glucose tolerance. Am. J. Obstet. Gynecol..

[33] Retnakaran R (2008). Glucose intolerance in pregnancy and future risk of pre-diabetes or diabetes. Diabetes Care.

[34] Hakkarainen H (2015). Post-challenge glycemia during pregnancy as a marker of future risk of type 2 diabetes: A prospective cohort study. Gynecol. Endocrinol..

[35] Retnakaran R (2008). Isolated hyperglycemia at 1 hour on oral glucose tolerance test in pregnancy resembles gestational diabetes mellitus in predicting postpartum metabolic dysfunction. Diabetes Care.

[36] Bo S (2006). Mild gestational hyperglycemia and the metabolic syndrome in later life. Metab. Syndr. Relat. Disord..

[37] Roeckner, J. T., Sanchez-Ramos, L., Jijon-Knupp, R. & Kaunitz, A. M. Single abnormal value on 3-hour oral glucose tolerance test during pregnancy is associated with adverse maternal and neonatal outcomes: A systematic review and metaanalysis. *Am. J. Obstet. Gynecol.* **215**, 287–297 (2016). 10.1016/j.ajog.2016.04.040 27133007

[38] Kokanali MK, Tokmak A, Kaymak O, Cavkaytar S, Bilge U (2014). The effect of treatment on pregnancy outcomes in women with one elevated oral glucose tolerance test value. Ginekol Pol..

[39] Langer O, Anyaegbunam A, Brustman L, Divon M (1989). Management of women with one abnormal oral glucose tolerance test value reduces adverse outcome in pregnancy. Am. J. Obstet. Gynecol..

[40] Hakkarainen H (2016). The risk of metabolic syndrome in women with previous GDM in a long-term follow-up. Gynecol. Endocrinol..

[41] Berezowsky A (2022). Glucose tolerance test with a single abnormal value in pregnancy and the risk of type-2 diabetes mellitus. Arch. Gynecol. Obstet..

[42] Kim KS, Hong S, Han K, Park CY (2021). The clinical characteristics of gestational diabetes mellitus in Korea: A National Health Information Database Study. Endocrinol. Metab. (Seoul)..

[43] Victoria R (2022). Perinatal outcomes in obese women with one abnormal value on 3-hour oral glucose tolerance test. Am. J. Perinatol..

